# The impact of confirmation bias awareness on mitigating susceptibility to misinformation

**DOI:** 10.3389/fpubh.2024.1414864

**Published:** 2024-10-15

**Authors:** Michal Piksa, Karolina Noworyta, Aleksander Gundersen, Jonas Kunst, Mikolaj Morzy, Jan Piasecki, Rafal Rygula

**Affiliations:** ^1^Affective Cognitive Neuroscience Laboratory, Maj Institute of Pharmacology Polish Academy of Sciences, Krakow, Poland; ^2^Department of Psychology, University of Oslo, Oslo, Norway; ^3^Faculty of Computing and Telecommunications, Poznan University of Technology, Poznan, Poland; ^4^Department of Philosophy and Bioethics, Faculty of Health Sciences, Jagiellonian University Medical College, Krakow, Poland

**Keywords:** fake news, intervention, confirmation bias, attitudes, vaccines, COVID-19, online research

## Abstract

**Introduction:**

In the current digital age, the proliferation of misinformation presents a formidable challenge to a democratic society. False narratives surrounding vaccination efforts pose a significant public health risk. Understanding the role of cognitive biases in susceptibility to misinformation is crucial in addressing this challenge. Confirmation bias, characterized by the tendency to favor information that aligns with pre-existing beliefs or attitudes, can exacerbate the spread of false narratives.

**Methods:**

This study investigates the effect of confirmation bias awareness on susceptibility to general misinformation. For this, a sample of 1,479 participants was recruited, ensuring diverse representation across attitudes towards vaccination. Half of the participants received targeted information about confirmation bias, aimed at increasing awareness of this bias and its potential impact on cognitive processing of information. The other half did not receive this information.

**Results:**

Results from the study indicated that participants exposed to an intervention aimed at inducing awareness of confirmation bias demonstrated reduced susceptibility to misinformation and increased ability to general discernment of veracity. Notably, these effects were only pronounced among individuals who initially were most negative towards COVID-19 vaccines.

**Discussion:**

These insights provide a foundation for developing targeted strategies to promote informed decision-making and mitigate the spread of misinformation, particularly in the context of public health crises. Further research is warranted to explore the underlying mechanisms driving these effects and to refine intervention approaches for diverse populations and contexts.

## Introduction

In an era marked by an unprecedented flow of information facilitated by digital technologies, the challenge of distinguishing fact from fiction has become increasingly daunting. Misinformation has permeated various aspects of society, ranging from politics (1) and public health (2) to economics (3) and culture (4). Its consequences are profound, undermining trust in institutions, eroding social cohesion, and even jeopardizing democratic processes (5). Particularly concerning is the proliferation of misinformation surrounding the safety of vaccines, with COVID-19 vaccines recently being a focal point of controversy (6). False narratives often question safety and efficacy of vaccines, labeling them as experimental or even deliberately engineered as bioweapons for public control purposes (7). Such polarizing misinformation not only undermines public trust in vaccination efforts but also poses a significant threat to global public health by fueling vaccine hesitancy and hindering efforts to curb the spread of epidemics. One of the serious consequences of this situation is the current outburst of infectious diseases that have been silent for decades, such as scarlatina, syphilis, lepra or measles (8).

### Cognitive mechanisms of susceptibility to misinformation

Given the severity of misinformation and its potentially life-threatening consequences, tackling it has become one of the paramount challenges (9). Research teams globally are investigating into various aspects of fake news, including misinformation mechanisms, individual susceptibility factors, and strategies to counter fake news dissemination (10). Multiple reports indicate that cognitive reflection, defined as the inclination to engage in analytical reasoning rather than relying solely on intuition (11), plays a crucial role in resilience against misinformation (12–14). Analytical reasoning involves critical thinking and logic in decision-making processes, contrasting with the default mode of intuitive reasoning which is prone to heuristics and subsequent cognitive biases (15).

### Cognitive biases

One of the examples of biased cognition, which is critical to examine within the realm of susceptibility to misinformation is confirmation bias (16). Its multifaceted nature can be concisely described as an unconscious inclination towards accepting those pieces of information that align with individual’s already existing beliefs or knowledge (17). This bias may manifest in various forms, such as selectively attending to corroborative information while disregarding incongruent data, and actively seeking out or favoring information that supports one’s preconceptions while avoiding or downplaying contradictory evidence (18).

Interestingly, many biases tend to lose their influence on information processing once individuals become aware of their existence (19). The same applies to confirmation bias, which has a significant role in contributing to susceptibility to misinformation (16, 20, 21). However, it is still uncertain whether increasing awareness about this bias could potentially lessen susceptibility to misinformation. By heightening awareness of confirmation bias, individuals are likely to activate their analytical reasoning systems, leading to increased vigilance in critically evaluating information sources and considering alternative viewpoints. They may develop strategies to actively counteract the bias, such as seeking out diverse perspectives or consciously engaging with conflicting evidence (22). Additionally, awareness of confirmation bias may prompt individuals to approach information congruent with their beliefs and knowledge with greater skepticism, leading to more careful scrutiny and analysis before accepting or spreading potentially misleading information, which aligns with the general concept of analytical and intuitive processing theory (15, 23). Research in the field of cognitive psychology and behavioral economics has shown that interventions aimed at increasing awareness of biases can indeed have a positive impact on decision-making processes (24). However, most of the research in the field of susceptibility to misinformation primarily concentrates on addressing misinformation directly (10), rather than exploring possible interventions that target the underlying mechanisms that contribute to susceptibility to misinformation. The present study goes beyond that symptomatic treatment, and instead, by investigating the impact of awareness of confirmation bias on susceptibility to misinformation, particularly in the context of attitudes towards COVID-19 vaccines, it tackles the causality of the problem. Ultimately, vaccine hesitancy is inherently intertwined with susceptibility to misinformation (25), positioning antivaccine beliefs as a predisposing factor towards susceptibility to misinformation and vice versa.

### Hypothesis

The central hypothesis of this experiment proposed that raising awareness of confirmation bias would bolster resistance to misinformation. Furthermore, the initial categorization of groups according to their opinions on COVID-19 vaccines, juxtaposed with susceptibility to misinformation assessed through a topic-neutral test, provides valuable insights into how susceptibility to misinformation in one subject area can be extrapolated to susceptibility in other areas.

## Methods

### Ethics statement

This study was conducted in accordance with the guidelines laid down in the Declaration of Helsinki, and all procedures involving research study participants were approved by the Bioethics Committee of Regional Medical Chamber in Krakow, Poland (L.dz.OIL/KBL/2/2023, from 1 February 2023). Informed consent was obtained from all participants.

### Participants

In total, 1,479 participants (*M_age_* = 41.51, *SD* = 13.13; women = 774, men = 678, non-binary = 27,) were recruited via Prolific Academic^1^, a platform with a worldwide pool of participants, that allows to recruit a specific sample, based on their prescreened characteristics. Two of the inclusion criteria were chosen – location (United States), and COVID-19 vaccine opinions, based on the prescreened characteristics:

- Positive (I feel positively about the vaccines; *n* = 497, PAG).
- Neutral (I do not have strong opinions either way; *n* = 492, NeuAG).
- Negative (I feel negatively about the vaccines; *n* = 490, NegAG).

The calculated sample sized indicated that considering unlimited population, at least 385 participants should reveal results with 95% confidence level, 5% margin of error.

**Figure 1:**
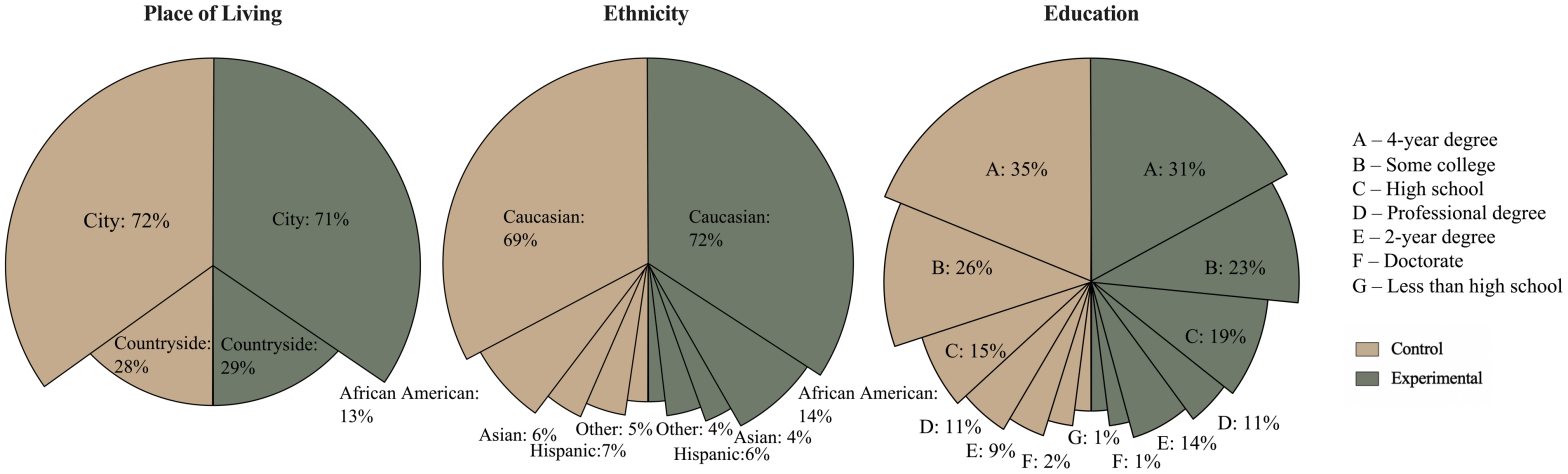
Demographical characteristics of control (left half) and experimental (right half) groups. The graphs present percentage distribution in control and experimental group separately.

This stratified sampling was followed to ensure that opponents, proponents, and those neutral of vaccines were equally represented, allowing for a reliable comparison of the effectiveness of the intervention between these groups. The participants had to confirm their opinions about the vaccines at the beginning of the survey. In case the opinions did not match the information provided to Prolific, the users could not participate in the survey any longer. All of the participants had to pass two attention checks (the same command in two different locations: *you must pay attention to this study. Please tick “X”*), and all participants answered all checks correctly. This was implemented to ensure the participants paid attention throughout the study. The participants were randomly assigned to one of two versions of the survey: experimental group (EG; *n* = 749; see procedure section), control group (CG; *n* = 730). More details about participants are presented in Figure 1.

### Procedure

**Figure 2:**
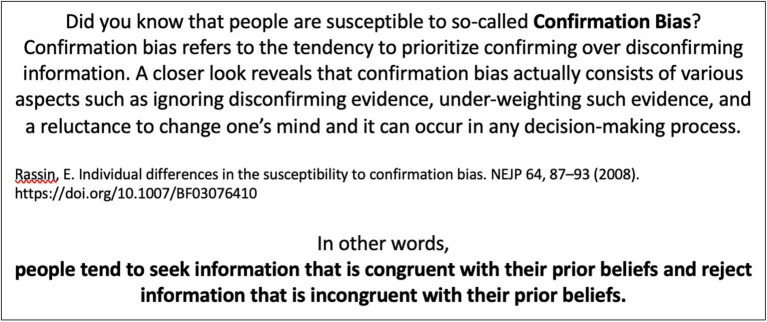
The information presented to EG aimed at induction of confirmation bias awareness.

The study was conducted between August 14th and September 25th, 2023. Following informed consent, the participants were redirected to the online testing platform *https://Qualtrics.com* , where they completed the survey. First, a baseline confirmation bias was measured using the Confirmation Bias Inventory (17) – a questionnaire consisting of 10 statements (e.g., *My first impression usually seems to be correct*) rated on a 5-point Likert scale from *strongly disagree* to *strongly agree* (Cronbach’s *α* = 0.83). The purpose of this inventory is to measure one’s proneness to confirmation bias (as a sum of scores from 10 items), defined as *the tendency to prioritize confirming over dis-confirming information* (17). The participants were then randomly assigned to one of two versions of the survey – one containing information raising awareness of confirmation bias (experimental group – EG; *n* = 749; see Figure 2), and another without such an intervention (control group – CG; *n* = 730).

Next, participants completed the Misinformation Susceptibility Test (MIST) (26). The MIST consists of 20 statements, which the participants were supposed to categorize as either true or false. The statements (10 true – e.g., *Democrats More Supportive than Republicans of Federal Spending for Scientific Research*; and 10 false – e.g. *Ebola Virus “Caused by US Nuclear Weapons Testing”, New Study Says*) were presented in random order and tackled different topics, from health and politics to economics. The test measures the ability to recognize true news (number of correctly assigned true news, Cronbach’s *α* = 0.75), fake news (number of correctly assigned false news; Cronbach’s α = 0.66), and a general ability to discern truthfulness (as a sum of both true and fake news recognition) (26). While the CG received an original instruction for this test (*Please categorize the following news headlines as either “Fake News” or “Real News”. Some items may look credible or obviously false at first sight, but may actually fall in the opposite category. However, for each news headline, only one category is correct*), the instruction for the EG, in addition, included the statement, “*Please, keep in mind that people tend to seek information that is congruent with their prior beliefs and reject information that is incongruent with their prior beliefs (Confirmation Bias).*”

The survey in addition consisted of demographic questions (i.e., gender, age, ethnicity, place of living, education level). After completing the survey, the participants were compensated with 4.50 GBP.

### Statistical analysis

The data were analyzed using GraphPad Prism for macOS (Version 10.1.1 (270), November 21, 2023). Differences between the EG and CG were assessed using an independent samples t-test (comparing control and experimental groups). One-way ANOVA was employed to examine differences in attitudes towards COVID-19 vaccines, while two-way ANOVA was utilized to explore interactions between vaccine attitudes and experimental manipulations. Šídák’s adjustment for multiple comparisons test was applied. Cohen’s d was calculated to gauge the effect sizes.

## Results

### Comparison of susceptibility to true and fake information, and proneness to confirmation bias between COVID-19 vaccine opinions groups

**Figure 3:**
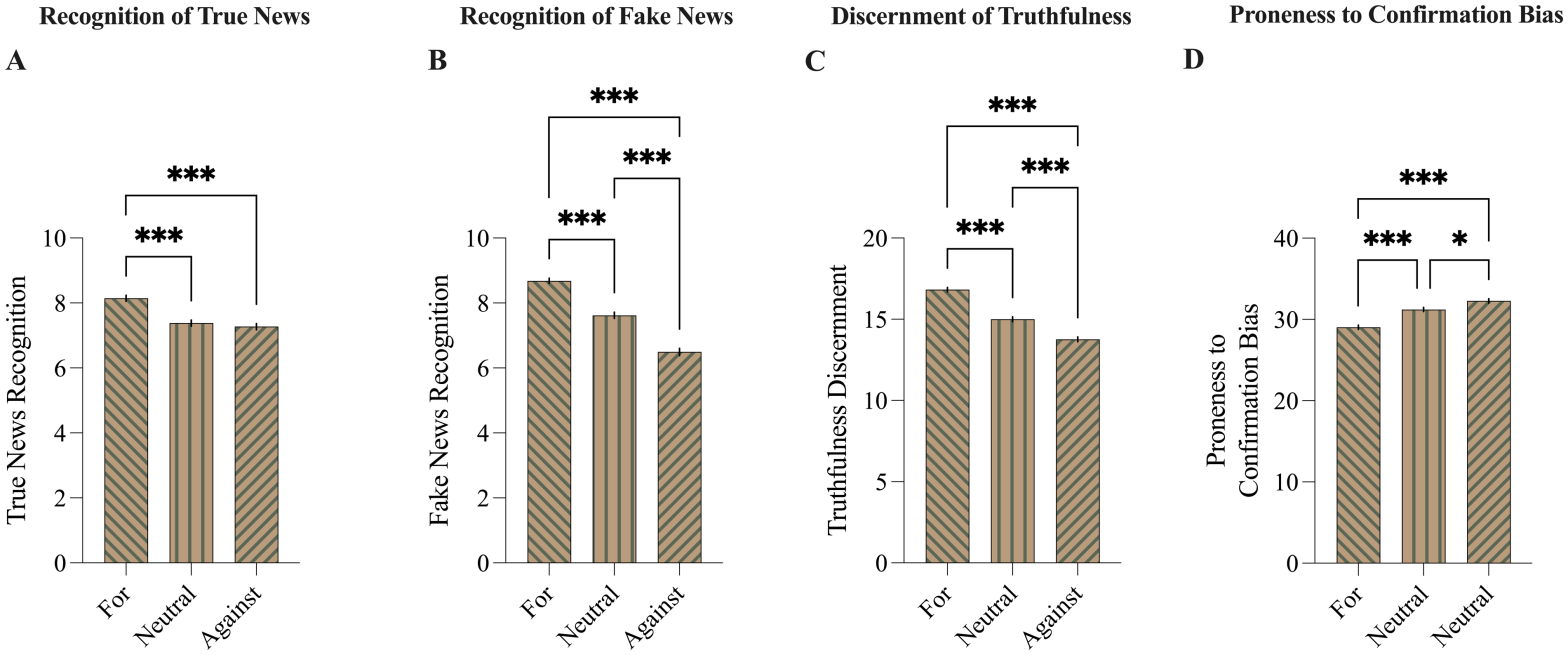
Comparison of susceptibility to true and fake news, and proneness to confirmation bias between COVID-19 vaccine opinions groups. Participants, categorized according to their opinions about COVID-19 vaccines, differed in their abilities to recognize **(A)** true news, **(B)** fake news, and **(C)** discernment of truthfulness. Additionally, variations were observed in their susceptibility to confirmation bias **(D)** – those opposed to vaccines were most prone to this bias. Means are presented with ± SEM; *p < 0.05, ***p < 0.001.

The groups distinguished based on participants’ attitudes towards COVID-19 vaccines differed in the ability to recognize true news (*F*_(2, 1,476)_ = 25.39, *p* < 0.001; Figure 3A). Additional post-hoc analysis revealed that PAG scored higher than NeuAG (*d = 0.*371, *p* < 0.001), PAG scored higher than and NegAG (*d = 0.*414, *p* < 0.001), but no significant difference between NeuAG and NegAG (*d = 0.*182, *p* =. 802).

These groups differed also in the ability to recognize fake news (*F*_(2, 1,476)_ = 131.19, *p* < 0.001; Figure 3B). Post-hoc analysis revealed that PAG scored higher than NeuAG (*d = 0.549*, *p* < 0.001), PAG scored higher than NegAG (*d =* 1.029, *p* < 0.001), and NeuAG scored higher than NegAG (*d* = 0.494, *p* < 0.001).

Moreover, the groups significantly differed in truthfulness discernment (*F*_(2, 1,476)_ = 114.2, *p* < 0.001; Figure 3C). Post-hoc analysis revealed that PAG scored higher than NeuAG (*d = 0.589*, *p* < 0.001), PAG scored higher than NegAG (*d = 0.951*, *p* < 0.001), and NeuAG scored higher than NegAG (*d* = 0.375, *p* < 0.001).

The groups also differed in their base level of proneness to confirmation bias (F_(2, 1,476)_ = 38.3, *p* < 0.001; Figure 3D). Additional post-hoc analysis revealed that PAG scored lower than NeuAG (*d = 0.*365, *p* < 0.001), PAG scored lower than NegAG (*d = 0.*537, *p* < 0.001), NeuAG scored lower than NegAG (*d = 0.*182, *p* = 0.017).

### Comparison of susceptibility to true and fake news between the control and experimental groups

**Figure 4:**
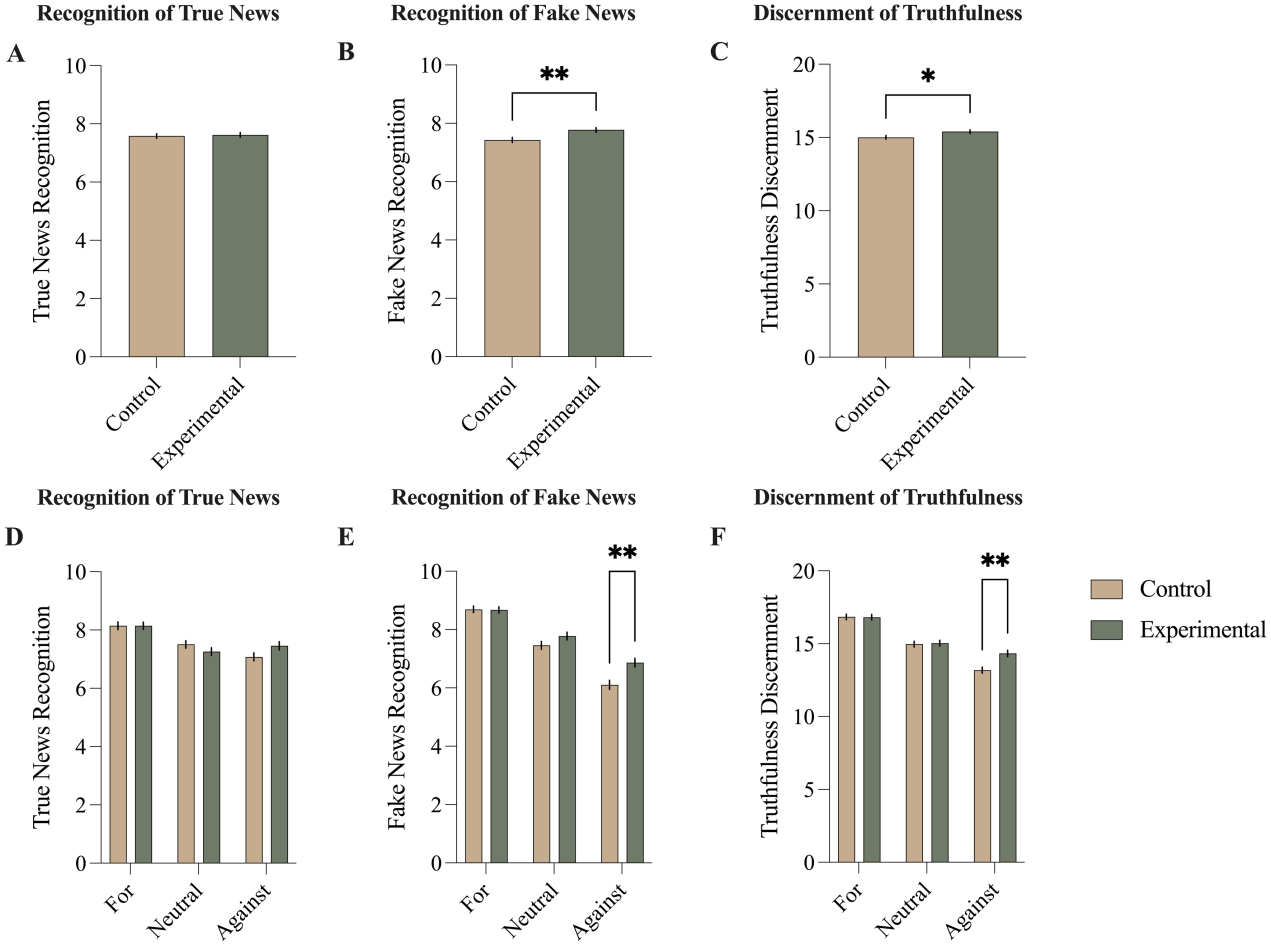
The impact of confirmation bias awareness on susceptibility to true and fake news. The control and experimental groups did not significantly differ in the average recognition of true news **(A)**. Significant differences were observed in the recognition of fake news **(B)** and discernment of truthfulness **(C)**. Adding the attitude towards COVID-19 vaccines factor, resulted in no significant interactions in recognition of true news **(D)**. However, the experimental group with a negative attitude towards COVID-19 vaccines, presented better recognition of fake news **(E)** and a higher ability to discern truthfulness **(F)**, when compared to the control group with such an attitude. Mean ± SEM; * *p* < 0.05 ** *p* < 0.01.

The CG and EG did not significantly differ in the average recognition of true news (t_(1477)_ = 0.3606, *d = 0.*019, *p* = 0.719; Figure 4A). EG was significantly better at recognizing fake news (t_(1477)_ = 2.898, *d = 0.*151, *p* = 0.003; Figure 4B) and discerning truthfulness than CG (t_(1477)_ = 2.157, *d = 0.*113, *p* = 0.031; Figure 4C).

### Comparison of susceptibility to true and fake news between the control and experimental groups distinguished by the attitudes towards COVID-19 vaccines

There were no significant interactions between the experimental groups and attitudes in the average recognition of true news (*F*_(2, 1,473)_ = 2.831, *p* = 0.059; Figure 4D). Significant interactions between these two factors were observed in the average recognition of fake news (F_(2, 1,473)_ = 4.202, *p* = 0.015; Figure 4E) and average truthfulness discernment (F_(2, 1,473)_ = 5.031, *p* = 0.007; Figure 4F). *Post hoc* multiple comparisons revealed that the differences between the control and experimental groups were only significant in the subgroups of NegAG (EG scored higher than CG in recognition of fake news: *d = 0.*313*, p* = 0.001; EG scored higher than CG in discernment of truthfulness: *d = 0.*342, *p* = 0.001; no difference in recognition of true news: *p* = 0.482). There were no significant differences between EG and CG in the PAG (recognition of true news, *p* > 0.999; recognition of fake news, *p* > 0.999; truthfulness discernment, *p* > 0.999), or NeuAG (recognition of true news, *p* = 0.957; recognition of fake news, *p* = 0.761; truthfulness discernment, *p* > 0.999).

## Discussion

The study employed an on-line experimental design to investigate the impact of awareness of confirmation bias on susceptibility to misinformation. The findings revealed that participants who received information about confirmation bias demonstrated increased resilience to misinformation. Furthermore, this effect was particularly pronounced among participants who held negative attitudes towards misinformation.

### Preliminary findings

The experiment began by assessing individual proneness to confirmation bias. Participants with a negative attitude exhibited higher levels of proneness to confirmation bias compared to those with neutral or positive attitudes (Figure 3D). Additionally, individuals with neutral attitudes scored higher on confirmation bias proneness compared to those with positive attitudes. The findings suggest that individuals who harbor skepticism or opposition towards vaccination may be more susceptible to confirmation bias, potentially reinforcing pre-existing beliefs or attitudes when exposed to fake news (27). Conversely, individuals with more positive attitudes towards vaccination may demonstrate lower levels of confirmation bias proneness, indicating a greater criticism towards misinformation. The nature of confirmation bias and misinformation suggests this is a circular interrelation with one factor powering another (28–30).

Likewise, the groups exhibited disparities in susceptibility to true news (Figure 3A), fake news (Figure 3B), and discernment of truthfulness (Figure 3C). Individuals with positive attitudes towards COVID-19 vaccines demonstrated the lowest susceptibility to general misinformation, while those with negative attitudes scored the highest, with the neutral opinions group falling between the extremes. One can notice that vaccine hesitancy can be caused by factors other than susceptibility to misinformation, such as previous experiences of side effects. However, the mentioned above results, prove that different attitudes towards COVID-19 vaccines were related to susceptibility to misinformation. Moreover, this observation aligns with the notion that susceptibility to misinformation on one theme correlates with susceptibility to misinformation on other related topics (31).

### Primary findings

The primary finding of this study underscores the effectiveness of inducing awareness of confirmation bias in reducing susceptibility to misinformation. Specifically, participants who received the information about confirmation bias exhibited lower susceptibility to fake news compared to the control group (Figure 4B). Interestingly, this effect was not accompanied by a significant difference in the recognition of true news between the experimental and control groups (Figure 4A). Moreover, participants in the experimental group demonstrated enhanced discernment of both fake and true news, indicating an overall improvement in their ability to differentiate between accurate and misleading information (Figure 4C). This suggests that raising awareness of confirmation bias not only mitigates susceptibility to misinformation but also enhances individuals’ capacity to critically evaluate the credibility of news.

The additional exploration of the effects observed in the study revealed an interesting moderation by attitude towards COVID-19 vaccines. Specifically, when attitude towards COVID-19 vaccines was included as a second factor in the analysis, significant differences in susceptibility to misinformation were only evident among individuals holding a negative attitude towards vaccination (Figures 4D–F). This finding aligns with the earlier observation that individuals with a negative attitude scored highest in baseline confirmation bias proneness (Figure 3). Analogously, this moderation effect can be understood through the lens of addressing the root cause of the problem. Just as there is no point in treating a sore throat if there is no sore throat present, interventions targeting confirmation bias would not be very effective among individuals who do not exhibit a predisposition towards confirmation bias or susceptibility to misinformation.

### Previous research

The findings presented in this study both align with and extend previous research in the domain of susceptibility to misinformation. Existing literature has consistently highlighted the role of cognitive reflection, an amalgamation of analytical and intuitive reasoning models (often referred to as systems 1 and 2), in bolstering resilience against misinformation (32). Within this framework, confirmation bias is a component of intuitive information processing (23). Prior studies have attempted interventions aimed at favoring intuitive over analytical thinking, such as manipulating response time limits, yielding successful outcomes in terms of reducing susceptibility to misinformation (33). However, to date, no research has specifically targeted confirmation bias itself, as undertaken in the current study. By directly addressing confirmation bias through a subtle experimental manipulation, this research contributes a new perspective to the literature and underscores the importance of exploring targeted interventions in the realm of misinformation susceptibility.

### Limitations

It is worth noting that the relatively small to moderate effect sizes observed in this study most likely resulted from the subtlety of the utilized intervention. One could hypothesize that employing a more substantial intervention, such as comprehensive psychological training aimed at addressing confirmation bias, might yield more pronounced effects. Such training could be more immersive and prolonged, potentially leading to deeper and more sustained cognitive restructuring (34). Future research could explore a balance between the intensity of the intervention and the duration of its implementation to better understand how to maximize both the potency and practicality of these psychological strategies.

There is one important point regarding the complexity of vaccine hesitancy, which extends beyond mere misinformation. While the present study focused primarily on negative attitudes towards COVID-19 vaccines as a proxy for susceptibility to vaccine misinformation, we acknowledge that this is a limitation. Negative attitudes towards vaccines can indeed stem from a variety of sources, including negative personal experiences with vaccines, concerns about side effects, or broader distrust in medical institutions. Concededly, presented results prove that different attitudes towards COVID-19 vaccines were related to general susceptibility to misinformation. However, the assumption that all negative attitudes equate to misinformation may oversimplify the diverse psychological factors at play. Future research should incorporate more stratified measures that differentiate between those who are misinformed and those who have vaccine hesitancy due to other reasons. Despite this limitation, the present findings still provide valuable insights into the broader trends in vaccine skepticism, though they should be interpreted with caution.

Unfortunately, the presented results also leave unanswered question regarding the potential longevity of the intervention’s effects. Previous work showed that people forget that something is misinformation fairly quickly (35). We do agree that knowledge or memory of what is true or false can be elusive. Yet, we do not know if awareness of cognitive mechanisms, such as cognitive bias, is comparable to knowledge or memory of facts. While it is plausible that once individuals become aware of their cognitive biases, they may maintain heightened awareness in the future, it is essential to consider the broader context of general reasoning models (36). According to this perspective, heuristics and biases are inherent to the default mode of processing information. Thus, the efficacy of interventions targeting confirmation bias may be constrained by the persistent tendency towards this default cognitive homeostasis. Future research endeavors should therefore aim to investigate the durability of intervention effects over time and explore strategies for sustaining awareness and mitigating biases in the long term.

## Conclusion

In conclusion, this study highlights the role of awareness of confirmation bias in mitigating susceptibility to misinformation, particularly in the context of attitudes towards COVID-19 vaccines. The findings demonstrate that individuals who received information about confirmation bias exhibited lower susceptibility to fake news, while still maintaining their ability to recognize true news. Moreover, the observed effects were moderated by attitudes towards COVID-19 vaccines, with significant differences in misinformation susceptibility observed primarily among individuals holding negative attitudes. These results underscore the importance of tailored interventions that address cognitive biases within specific subgroups to effectively combat misinformation and promote informed decision-making. By understanding the nuanced interplay between individual characteristics and cognitive vulnerabilities, public health efforts can be better directed towards populations where interventions are most needed, ultimately enhancing the dissemination of accurate information and mitigating the harmful effects of misinformation.

## Data Availability

The datasets presented in this study can be found in online repositories. The names of the repository/repositories and accession number(s) can be found below: https://doi.org/10.17605/OSF.IO/JRKZE.

## Footnotes

^1^ https://prolific.com
